# Per- and poly-fluoroalkyl substances as forever chemicals in drinking water: Unraveling the nexus with obesity and endocrine disruption – A mini review

**DOI:** 10.1016/j.heliyon.2025.e42782

**Published:** 2025-02-18

**Authors:** Hoda Pezeshki, Saeed Rajabi, Majid Hashemi, Saeideh Moradalizadeh, Habibeh Nasab

**Affiliations:** a Environmental Health Engineering Research Center, Kerman University of Medical Sciences, Kerman, Iran; b Student Research Committee, Kerman University of Medical Sciences, Kerman, Iran; c Student Research Committee, Shiraz University of Medical Sciences, Shiraz, Iran; d Department of Environmental Health Engineering, School of Health, Shiraz University of Medical Sciences, Shiraz, Iran; e Student Research Committee, Shahid Sadoughi University of Medical Sciences, Yazd, Iran; f Environmental Science and Technology Research Center, Department of Environmental Health Engineering, School of Public Health, Shahid Sadoughi University of Medical Sciences, Yazd, Iran

**Keywords:** Endocrine disrupting, Obesity, PFASs, Potable water

## Abstract

Per- and Poly-fluoroalkyl substances (PFASs) are among the substances that have been widely employed across the world due to their distinct features. These chemicals' great stability in the environment and capacity to be released from consumer goods have demonstrated their existence in all matrices. Additionally, the world's attention has been drawn to these substances' direct relation to human health in recent years. This research aimed to unravel the nexus of PFASs with obesity and endocrine disruption as a comprehensive review. Studies have shown that drinking water is the primary way in which humans are exposed to PFASs. As a result, it has become difficult to determine how drinking water is contaminated with these compounds and how their impacts affect human health. Through various routes, disruption of metabolic processes, and possible effects on the hypothalamus-pituitary-thyroid axis, these chemicals increase the incidence of obesity, particularly during crucial growth phases. Another negative health impact of PFASs is the disruption of the endocrine glands' function, which is crucial for controlling the body's physiological functions. This leads to alterations in hormone production. The effects of exposure to these substances include secondary hyperparathyroidism, non-alcoholic fatty liver, diabetes, cardiovascular illnesses, reproductive abnormalities, and infertility. Because of their characteristics, including the propensity to propagate through the food chain, accumulate and biomagnify, and ultimately pose a threat to human life, it is crucial to replace and remove these chemicals.

## Highlights

• PFASs are widely used globally due to their unique features and release from consumer goods.
• Recently focused on PFASs' direct impact on human health, particularly via drinking water exposure.
• PFASs disrupt metabolic processes, the thyroid axis, and endocrine glands, leading to obesity and reproductive issues.

## Graphical abstract

**Figure:**
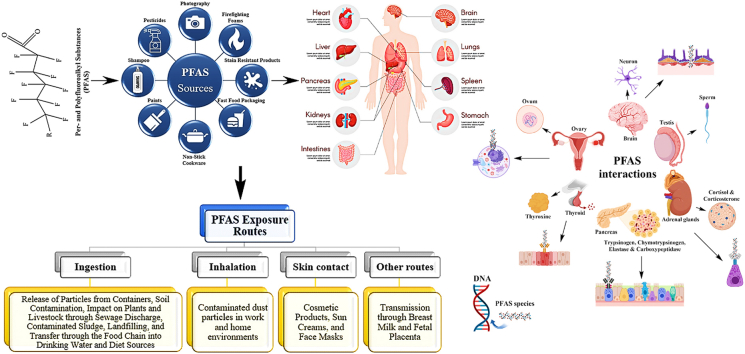

## 1 Introduction

Given the direct influence of healthy nutrition and, paramountly, clean drinking water on human health, ensuring access to and safeguarding the quality of potable water stands as an indispensable cornerstone in the preservation and sustenance of human life [1,2]. The burgeoning global population and escalating industrial and agricultural endeavors have surged water consumption rates. Concurrently, these activities have brought a slew of new contaminants into water sources, resulting in a significant deterioration in water quality in recent years [3]. Per the World Health Organization (WHO), nearly 10 % of the global populace lacks access to secure and potable water sources [4]. Furthermore, a staggering 6 % of fatalities in underdeveloped nations stem from the consumption of contaminated water, underscoring the dire impact of inadequate access to clean drinking water [5]. Hence, ensuring abundant access to healthy drinking water stands as a pivotal factor in mitigating mortality rates. So much so that the provision of safe drinking water has emerged as a crucial goal within the United Nations' agenda [6].

Nevertheless, insufficient awareness regarding the environmental and health impacts of synthetic compounds coupled with their rampant usage, alongside the daily discharge of approximately 2 million tons of pollutants into water bodies, has resulted in widespread contamination of global water resources [[7], [8], [9], [10]]. The emergence of various pollutants including pharmaceutical compounds, endocrine-disrupting chemicals, perfluorinated compounds, disinfection by-products, pesticides, microplastics, and more, poses a significant threat to both environmental integrity and human well-being [3,11].

Contaminants of Emerging Concern (CECs) encompass a diverse spectrum of chemical substances that have garnered attention due to their potential to adversely impact environmental ecosystems and human health [12]. These contaminants infiltrate environmental matrices via multiple pathways, including discharge from wastewater treatment plants, agricultural runoff, industrial effluents, and atmospheric deposition. Their resilience to degradation and persistent introduction into natural systems have sparked apprehensions about their enduring influence on ecological equilibrium and human health [13,14].

A pivotal facet of CECs lies in their ability to elicit biological effects even at remarkably low concentrations, often surpassing conventional toxicity thresholds for known contaminants [13]. This low-dose impact raises alarm regarding their potential cumulative repercussions over time, encompassing concerns such as endocrine disruption, genotoxicity, and the proliferation of antibiotic resistance among microorganisms [12,14]. Of these, per- and poly-fluoroalkyl substances (PFASs), classified as emerging pollutants within the realm of CECs, have notably contaminated drinking water sources in recent times [13].

All the traditional methods of environmental monitoring, such as laboratory analysis and discrete sampling, are all burdened by high costs, intensive time use, and inability to delineate the dynamic nature of the chemical species in aquatic environments. They also face challenges in maintaining sample stability over longer periods. High Performance Liquid Chromatography-Mass/Mass (HPLC-MS/MS) with electrospray ionization is applied to identify ionic PFAS [15], while neutral compounds are usually analyzed by Gas Chromatography-Mass (GC-MS) [16]. Despite their high sensitivity and specificity, both methods are costly and complicated. Real-time, continuous monitoring techniques such as electrochemical sensors have been developed to overcome the above limitations. These sensors are promising for on-site PFAS detection with high temporal and spatial resolution, owing to their low cost, portability, and very high sensitivity [17,18]. Despite the promise of electrochemical and opto-electrochemical sensors, there are some drawbacks related to selectivity issues and interference by other substances. Other techniques, such as Molecularly Imprinted Polymers (MIPs) and Surface-Enhanced Raman Spectroscopy (SERS), also show promise due to their high sensitivity and specificity, though they require sophisticated equipment or optimization [19,20].

## 2 Per- and poly-fluoroalkyl substances (PFAS)

Per- and Poly-fluoroalkyl substances (PFASs) also mentioned “Forever Chemicals” constitute a group of fluorinated chemicals originating from late 1940s development, gaining substantial usage across industries and commerce since the late 1960s [[21], [22], [23]]. PFAS molecules exhibit a dual nature, characterized by hydrophobic and hydrophilic ends, featuring functional groups such as carboxylic (R-COOH), sulfonate (R-OSO_2_), and various other functional moieties [24]. Perfluoroalkyl compounds constitute fluorinated aliphatic substances wherein carbon-hydrogen bonds are entirely substituted by carbon-fluorine bonds. Conversely, in polyfluoroalkyl materials, this substitution is not entirely comprehensive, allowing for the presence of some hydrogen atoms alongside fluorine [25,26]. The robustness of PFAS compounds stems from the strength of the C-F bond, recognized as one of the most resilient covalent bonds, ensuring their thermal stability. Moreover, the incorporation of terminal functional groups along the fluoroalkyl chain contributes significantly to the chemical stability of these compounds [24,27]. Within PFASs, distinctions arise between two prominent groups: Perfluoroalkyl Carboxylic Acids (PFCAs) and Perfluoro Sulfonic Acids (PFSAs), delineated by variations in the carbon chain length and the terminal functional group [27]. The bioaccumulation and biomagnification tendencies of PFSAs exhibit an upward trend alongside an increase in carbon chain length. Conversely, PFCAs of similar chain lengths demonstrate a higher propensity for bioaccumulation [27]. Perfluoro Octanoic Acid (PFOA), a member of the PFCA group, and Perfluoro Octane Sulfonate (PFOS), belonging to the PFSA category, stand out as among the most recognized and extensively utilized PFAS compounds [28].

The exceptional hydrophobicity and lipophobicity, coupled with their remarkable resistance to thermal, biological, and chemical degradation, as well as oxidative stability, render PFAS compounds highly sought-after for a myriad of applications. Their unique traits have propelled their utilization as surfactants, lubricants, and additives across diverse industries encompassing textiles, electronics, food packaging, household products, cosmetics, pharmaceuticals, medical equipment, and pesticides. Moreover, PFAS compounds contribute to the manufacturing of waterproof fabrics, firefighting foams, masks, photographic films, and various other goods [22,25,27,28]. Nevertheless, the extensive utilization of PFAS compounds, coupled with their notable environmental persistence and intrinsic traits such as bioaccumulation and high mobility, results in their release from diverse sources. Consequently, these substances contaminate the food chain, precipitating pervasive environmental pollution. Human exposure occurs through three primary routes: inhalation, ingestion, and skin contact, contributing significantly to the widespread exposure of humans to these compounds [22,24,[29], [30], [31]].

Drinking water stands out as the primary exposure pathway for this pollutant, particularly in communities grappling with water affected by various factors, including industrial effluent discharge from fluorochemical production, utilization of PFAS-based firefighting foams, and sewage treatment plant effluents [29,32]. Per the 2018 guidelines by the WHO, the recommended limit for the overall concentration of PFAS in water stands at 0.5 μg/L, with a further stipulation of 0.1 μg/L as the recommended limit for each compound within this group [27]. The standards established by various international organizations concerning PFAS compounds are detailed in Table 1. Following consumption, PFAS compounds undergo minimal metabolism and excretion, persisting within the human body for approximately 5–8.5 years [33].

**Table 1:** Drinking water standards for PFAS compounds across several countries.

Standards regulated organizations	Value	Ref.
US Environmental Protection Agency	PFOA: 0.07 μg/L	[22]
UK Health Protection Agency	PFOS: 0.3 μg/L	[22]
	PFOA: 10 μg/L	
Australian Department of Health	PFOS & PFHxS: 70 ng/L	[25]
	PFOA: 560 ng/L	
German Commission	PFOS & PFOA: 0.1 μg/kg BW	[22]
European Commission	Total PFAS: 500 ng/L	[25]
Health of Canada	PFOS: 600 ng/L	[160]
	PFOA: 200 ng/L	
Italy	PFOS: ≤30 ng/L	[22]
	PFOA: ≤500 ng/L	
	Other PFASs: ≤500 ng/L	

**PFOA:** Perfluorooctanoic acid.

**PFOS:** Perfluorooctane sulfonate.

**PFAS:** Perfluoroalkyl substances.

**PFHxS:** Perfluorohexanesulphonic acid.

Exposure to these pollutants can profoundly impact health, manifesting in a range of severe disorders including endocrine disruptions, obesity, cardiovascular diseases, liver ailments, diabetes, fertility issues, thyroid dysfunction, musculoskeletal disorders, and potential carcinogenic effects [26,[33], [34], [35], [36]]. The intricate mechanisms of the endocrine system regulate numerous vital functions within the body. Any disruption or disorder within this system can significantly impact human health and well-being [33]. PFAS compounds, categorized as endocrine-disrupting chemical compounds (EDCs), pose a substantial threat to human health [36]. The production and utilization of certain variants like PFOA and PFOS, deemed persistent organic pollutants (POPs), have led to their prohibition across numerous countries due to their enduring presence and detrimental effects [29].

Per- and polyfluoroalkyl substances encompass a broad class of chemicals classified into two primary groups: polymers and non-polymers. Among these, non-polymer compounds have garnered heightened attention due to their pervasive detection in the environment [37]. Non-polymer compounds are further categorized into two distinct classes: perfluoroalkyl acids (PFAA), commonly referred to as perfluoroalkyl compounds and polyfluoroalkyl substances. Perfluoroalkyl compounds are characterized by fully fluorinated carbon chains, while polyfluoroalkyl materials are only partially fluorinated and may contain non-fluorine atoms such as hydrogen or oxygen [37,38]. In the context of this study, particular focus has been directed towards perfluorinated carboxylic acids (PFCAs) and perfluorinated sulfonic acids (PFSAs), which represent the principal subgroups within the realm of PFAAs under investigation [37]. These compounds, due to their unique chemical properties and widespread occurrence, pose significant challenges in terms of environmental persistence and potential adverse effects, thereby warranting comprehensive examination and analysis.

The extensive distribution and persistence of PFASs in different water sources across the world are shown in Table 2, highlighting serious health and environmental problems. From drinking water and groundwater to precipitation in far-flung places like the Arctic, PFAS contamination is seen in a variety of water matrices. In sharp contrast to the lower amounts (PFOA: 0.53 ng/L) found in Arctic precipitation [39], PFOA has been found in groundwater in the Veneto Region of Italy at concentrations as high as 1173 ng/L. This illustrated the twin problems of localized contamination from industrial processes and the worldwide movement of PFASs through hydrological and atmospheric pathways. These trends highlight how widespread PFAS contamination is, regardless of ecological or geographic isolation [22].

**Table 2:** Detected PFASs in different sources of water.

Water Sources	PFASs concentration	Ref.
Drinking Water of Veneto Region, Italy	PFOA: 500 μg/L	[22]
	PFOS: 30 ng/L	
	Other PFAS: 500 ng/L	
Precipitation/Snow at Arctic	PFNA: 0.45 ng/L	[39]
	PFOA: 0.53 ng/L	
Cornwallis Island Lake, Arctic	PFOA: 0.20–0.27 ng/L	[39]
Groundwater Under Landfills, Australia	PFAS: 26–5200 ng/L	[42]
	PFHxS: 2.6–280 ng/L	
	PFOS: 1.3–4800 ng/L	
	PFHxA: >46 ng/L	
	PFOA: 1.7–74 ng/L	
Natural Mineral Water, France	PFHxA: 139 ng/L	[44]
	PFAS: 6.7 ng/L	
Ruhr Catchment Area, Germany	PFOA: 519 ng/L	[161]
	PFHpA: 23 ng/L	
	PFHxA: 22 ng/L	
Drinking Water of Uppsala, Sweden	PFHxS: 25 ng/L	[40]
	PFOS: 45 ng/L	
Tap Water, Catalonia, Spain	PFOS: 0.39–0.87 ng/L	[41]
	PFOA: 0.32–6.28 ng/L	
Drinking Water of Rio de Janeiro State, Brazil	PFOS: 0.58–6.70 ng/L	[162]
	PFOA: 0.35–2.82 ng/L	
	PFHxS: 0.15–1.00 ng/L	
Wastewater Treatment Plants, China	PFOA: 18.4–41.1 ng/L	[45]
	PFOS: 1.69–3.85 ng/L	
Water Treatment Plants of Osaka, Japan	PFOS: 1.3 ng/L	[163]
	PFOA: 3.7 ng/L	
Groundwater of Veneto Region, Italy	PFOA: 1173 ng/L	[22]
	PFUnA: 640 ng/L	

**PFOA:** Perfluorooctanoic acid.

**PFOS:** Perfluorooctane sulfonate.

**PFUnA:** Perfluoroundecanoic acid.

**PFAS:** Perfluoroalkyl substances.

**PFHxS:** Perfluorohexanesulphonic acid.

**PFHxA:** Perfluorohexanoic acid.

**PFHpA:** Perfluoroheptanoic acid.

PFAS concentrations in drinking water differ globally. PFOS levels of 45 ng/L in Sweden [40] and a range of PFOS measurements (0.39–0.87 ng/L) in tap water from Catalonia, Spain are noteworthy instances. Even though these concentrations are lower than in industrial locations, they are nonetheless significant due to the potential of chronic exposure to populations of people [41]. Extreme PFAS fluctuation is seen in Australia's groundwater beneath landfills, with concentrations as high as 5200 ng/L, which is indicative of leachate contributions [42]. The groundwater in the Veneto Region also has elevated levels of PFOA and PFUnA, indicating that industrial effluents are a major source. These hotspots demonstrate the need for more stringent waste management procedures in order to stop leaking into subsurface water supplies [22,43]. PFASs are found in natural sources such as mineral water in France (PFHxA at 139 ng/L), most likely as a result of air deposition or infiltration from polluted soil [44]. In China and Japan, wastewater treatment plant effluents and other urban water sources exhibit residual PFASs, including PFOA (41.1 ng/L) and PFOS (1.3 ng/L) [45].

### 2.1 PFCAs

A subset of perfluoroalkyl acids (PFAAs) conforms to the chemical formula C_n_F_2n+1_-COOH and is further categorized into two distinct groups: long-chain PFCA compounds, characterized by more than seven carbons, and short-chain PFCA compounds, featuring less than seven carbons [26,46]. Notably, compounds with longer carbon chains exhibit a pronounced affinity for binding to particles and possess a heightened potential for bioaccumulation, whereas those with shorter chains are typically found in the aqueous phase [47]. Primarily utilized within the fluoropolymer industry, these compounds play a pivotal role in various applications. Remarkably, a significant proportion—approximately 80%—of PFCA compounds released into the environment stem directly from the production processes associated with fluoropolymers [26]. Notable examples of these compounds include perfluorooctanoic acid (PFOA), perfluorononanoic acid (PFNA), and perfluorodecanoic acid (PFDA) [48].

### 2.2 PFSAs

Another significant category of PFAAs conforms to the chemical formula C_n_F_2n+1_-SO_3_H, mirroring the structural characteristics of PFCA compounds. Similar to their PFCA counterparts, these PFAA compounds are subdivided into two primary groups: long-chain PFSA compounds, comprising more than seven carbons, and short-chain PFSA compounds, containing fewer than seven carbons [46,47,49]. As observed with PFCA compounds, those with longer carbon chains exhibit a heightened propensity for particle binding and bioaccumulation, while shorter-chain variants are typically encountered in the aqueous phase [46,47,49]. Notable examples within this category include perfluorooctane sulfonic acid (PFOS) and perfluorohexane sulfonic acid (PFHxS) [48].

### 2.3 PFOAs

Perfluorooctanoic acid (PFOA), with the chemical formula C_8_HF_15_O_2_, is a representative example of long-chain PFCA with a molecular weight of 414.07 g/mol. It is characterized by a melting range of 55–56 °C and an initial boiling point of 189 °C, with a vapor pressure of 0.69 hPa at 25 °C [50]. PFOA typically exists in the form of colorless flakes and predominantly as a neutral acid, exhibiting solubility in water across a wide range of pH values and primarily in its anionic form [28,37,51]. The versatility of PFOA finds application in various industrial sectors, including surfactants, food packaging, the production of Teflon, textiles, and firefighting foams. Additionally, it serves as a key component in water and oil repellants for fabrics, contributing to their enhanced performance characteristics [[52], [53], [54]].

### 2.4 PFOSs

Perfluorooctane sulfonic acid (PFOS), represented by the chemical formula C_8_HF_17_O_3_S, is a long-chain PFSA compound with a molecular weight of 500.13 g/mol [55,56]. Unlike PFOA compounds, PFOS is typically encountered in the form of a liquid with a distinctive bright red coloration. Similar to PFOA, PFOS predominantly exists as a neutral and soluble acid in water, demonstrating solubility across a wide pH range and primarily appearing in its anionic form [28,37,52]. The primary applications of PFOS compounds are notably observed in firefighting foams and textiles. Due to its unique properties, PFOS is valued for its effectiveness in firefighting applications and its ability to impart desirable characteristics to textiles, such as water and oil repellency [52].

### 2.5 PFHxS

Perfluorohexane sulfonic acid (PFHxS), characterized by the chemical formula C_6_HF_13_O_3_S, is categorized as a short-chain PFSA compound with a molecular weight of 400.11 g/mol [28,48,57]. Renowned for its physical and chemical stability, PFHxS serves as an effective alternative to longer-chain PFOS and PFOA compounds, particularly in applications where surfactants are required [57]. This compound finds widespread usage in various industries owing to its efficiency and versatility. In addition to its role as a surfactant, PFHxS is employed in firefighting foams and plays a crucial role in carpet and fabric protection applications [58]. Its chemical stability, combined with its surfactant properties, makes it a preferred choice in industries where durability and efficacy are paramount.

### 2.6 PFNAs

Perfluorononanoic acid (PFNA), represented by the chemical formula C_9_HF_17_O_2_, is classified as a long-chain PFCA compound, with a molecular weight of 464.08 g/mol [59]. It is characterized by a melting range of 59–62 °C and an initial boiling point of 218 °C. PFNA typically manifests in the form of beige crystals [28,51]. Primarily utilized as surfactants, PFNA compounds play a significant role in various industrial processes, particularly in the synthesis of textiles and polymers [52]. Their surfactant properties make them invaluable in facilitating the production and processing of these materials, contributing to improved performance characteristics and overall product quality.

### 2.7 PFDAs

Perfluorodecanoic acid (PFDA), with the chemical formula C_10_H_19_O_2_, belongs to the category of long-chain PFCA compounds. It has a molecular weight of 514.08 g/mol [60]. PFDA typically exists in the form of white powder, with a melting range of 77–81 °C and an initial boiling point of 218 °C [28,51]. PFDA compounds find diverse applications, with notable use in anti-stain and grease coatings applied to various materials, including food packaging, sofas, and carpets [52]. These compounds impart desirable properties such as water and oil repellency, enhancing the durability and cleanliness of the treated surfaces.

## 3 Application and transmission of PFAS in the environment and human systems

Given the widespread use and notable mobility of PFAS substances in various consumer products, their pervasive release has led to contamination in drinking water sources, air, and dietary sources. Consequently, human exposure occurs through three primary routes: inhalation, ingestion, and skin contact [21,30,61]. The release of waste, leachate, and sludge containing PFAS compounds has led to soil pollution, subsequently impacting plants. Through their cumulative properties and transmission via the food chain, these pollutants have further contaminated animals, highlighting the interconnected environmental impact of PFAS pollution [7,26,28,61,62]. Similarly, the discharge of contaminated sewage has resulted in the pollution of water sources, subsequently impacting marine life, particularly fish populations [32,36,63]. Furthermore, water source contamination has been notably observed in proximity to industrial facilities, military bases, airports, and fire stations, ultimately culminating in the contamination of drinking water supplies [37,62,63]. PFASs are intricately intertwined with the issue of global plastic pollution. While some PFAS compounds exist as microplastic particles, others transform into meso- or micro-plastics throughout their production, application, and disposal phases.

Additionally, microplastics, acting as carriers, contribute to the release of these compounds into the environment in certain instances [7]. Another significant pathway for releasing these compounds into the environment involves their discharge from textiles, food packaging, cooking utensils, and various other everyday objects [61,64]. The migration of these substances into food from containers is contingent upon several factors, including the pH level, humidity, fat content, and salt concentration of the food. Studies indicate that these compounds tend to exhibit higher release rates from containers holding alcoholic beverages, fatty foods, items with lower pH and moisture levels, and those with elevated salt content [65].

**Fig. 1:**
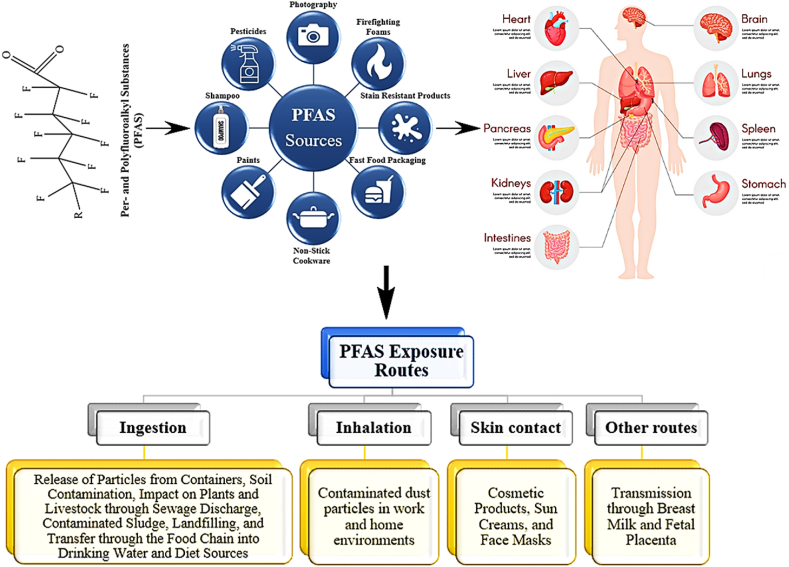
Exposure pathways of PFAS in the human body.

Occupational exposure is a crucial consideration, particularly in professions such as metal plating, firefighting, and textile manufacturing, where workers face potential risks through direct contact with materials and inhalation of PFAS-contaminated air [36,63,66,67]. These compounds have been incorporated into various products like cosmetics, sunscreens, and face masks, among others, resulting in human exposure primarily through skin adsorption [31,36]. Among the most vulnerable groups, infants are particularly impacted by this pollutant through exposure via the placenta during pregnancy and breastfeeding [68]. The primary pathways of exposure to this pollutant are through drinking water and diet. Once ingested, the human body eliminates a small amount of this pollutant through various mechanisms, while the remainder gets absorbed, binding to proteins in the blood and other tissues [36,61]. Over time, these compounds accumulate in various tissues such as the lungs, liver, kidneys, brain, and bones, leading to a diverse array of health issues [23]. Fig. 1 illustrates the pathways through which PFAS are exposed and enter the human body.

## 4 Instrumental analysis and PFASs measurement

Levels of PFASs in the different environmental matrices, especially in drinking water, determine what potential harmful exposure these compounds have to human health. Science-based toxicity evaluations develop regulatory limits of PFAS exposure, and more specifically, have been set in a chemical- and region-specific manner. Typical ranges for hazardous levels of PFAS range between part per trillion (ppt) to part per billion (ppb) [69]. Bioaccumulation of PFASs in human tissue and aquatic organisms gives rise to the need for continuous monitoring at ever-lower concentrations. Effective management and mitigation of the contamination require appropriate detection methods for PFASs. Various analytical techniques have their own advantages and disadvantages concerning the practicality, sensitivity, and specificity of detecting PFASs in water (Table 3) [70].

**Table 3:** PFASs detection techniques.

Compound	Detection Techniques	Advantages	Limitations
PFOA	GC-MS: 0.1–1 ng/L	High sensitivity, well-established, widely applicable	Expensive, time-consuming, complex sample prep for GC-MS
	HPLC-MS/MS: 0.1		
	Electrochemical Sensors: 0.1 ng/L		
PFOS	GC-MS: 0.1–1 ng/L	Commonly found, effective for detection	Limited for ionic PFASs in GC-MS
	HPLC-MS/MS: 0.1 ng/L		
	Electrochemical Sensors: 0.02 ng/L		
PFNA	GC-MS: 0.1–1 ng/L	Sensitive to environmental samples	Expensive, requires skilled operators for HPLC-MS/MS
	HPLC-MS/MS: 0.1 ng/L		
PFHxS	GC-MS: 0.1 ng/L	Can analyze a wide range of PFASs	Calibration needed, interference from other substances
	HPLC-MS/MS: 0.05 ng/L		
PFHxA	GC-MS: 0.1–1 ng/L	Reliable detection, highly sensitive	Requires pre-treatment for GC-MS
	HPLC-MS/MS: 0.011 ng/L		
PFHpA	GC-MS: 0.1–1 ng/L	Common in wastewater, reliable method	High cost, requires expertise
	HPLC-MS/MS: 0.01 ng/L		

**PFOA:** Perfluorooctanoic acid.

**PFOS:** Perfluorooctane sulfonate.

**PFNA:** Perfluorononanoic acid.

**PFHxS:** Perfluorohexanesulphonic acid.

**PFHxA:** Perfluorohexanoic acid.

**PFHpA:** Perfluoroheptanoic acid.

GC-MS is, up to now, a classic analytical technique for detecting neutral PFASs such as long-chain perfluorinated compounds like PFOA and PFOS. It has excellent sensitivity and selectivity, with the limits of detection ranging from 0.1 to 1 ng/L [71]. However, sample preparation for GC-MS normally is complicated, involving extraction and concentration, and thus usually requires much time and labor. Furthermore, it has shown less satisfactory effectiveness in analyzing ionic PFASs [72].

HPLC-MS/MS has been the golden standard in the detection of ionic PFAS due to its high sensitivity and specificity, detecting as low as 0.011–0.04 ng/L [73,74]. This method is, thus, particularly helpful in the case of chemicals like PFHxS and PFNA. HPLC-MS/MS is very useful in environmental monitoring because it can analyze a lot of PFASs in a single run. However, it is less viable for the normal on-site application process because this usually requires expensive equipment and skilled labor [75].

Novel electrochemical sensing methods have huge potential with regard to on-site real-time PFAS detection. These sensors detect PFASs through electrochemical mechanisms with the use of electrodes as transduction elements. Electrochemical sensors are compact, portable, and relatively inexpensive. They are highly sensitive and can be applied for continuous monitoring. These sensors proved to be especially helpful for field applications where sample transportation is not possible and, in such applications, can detect PFASs as low as 0.002–0.1 ng/L [20,76]. However, electrochemical sensors have a few major drawbacks: periodic calibration is often needed; there is interference from other substances that may interfere with the result; and selectivity problems regarding particular PFASs. By combining the advantages of optical sensing and electrochemical detection, opto-electrochemical sensors offer higher sensitivity and selectivity for PFASs. These sensors are suitable for continuous monitoring and provide real-time data with low power consumption. Because of the high specificity of the opto-electrochemical sensors, PFASs can be well detected even in a complicated environmental matrix [19,77]. However, they do require some basic equipment, and practical application may face interference from samples that could lower performance. Notwithstanding these advantages, issues of calibration errors and interference from other substances still exist, needing further research and enhancement to make the technologies more applicable for routine environmental monitoring. Most likely, multi-technique approaches with both established and state-of-the-art techniques will provide the most reliable and comprehensive solutions regarding the detection of PFAS in environmental studies [20].

Biosensors are analytical devices consisting of a biological recognition element coupled with a transducer; this is used for converting the biological response to a detectable signal. These devices will have the ability to offer fast, sensitive, and specific detection in a wide range of applications, including environmental monitoring, healthcare, and food safety. The main elements that compose biosensors include biological receptors, transducers, and signal processors [78]. Label-free electrochemical immunosensors, which are for the detection of PFASs, are based on specific antibodies or aptamers and thus generate electrochemical signals proportional to the concentrations of PFASs. They offer the advantages of high sensitivity, portability, and low cost; however, challenges with respect to specificity, interference, and stability remain [79,80].

## 5 Impact of PFAS on obesity and endocrine disruption

### 5.1 Obesity

As per the WHO, obesity, defined as a Body Mass Index (BMI) exceeding 30 kg/m^2^, refers to the abnormal accumulation of fat in various body regions, posing a significant health risk [81]. More precisely, obesity is characterized by the abnormal accumulation of body fat exceeding 20 % of total body weight, surpassing the threshold of the ideal body weight [82]. Recognized as a chronic condition, obesity stands as a pivotal contributor to the escalating prevalence of chronic and non-communicable diseases worldwide [[83], [84], [85]]. Since the late 20th century, the prevalence of obesity and associated health issues has surged dramatically. In 2016, approximately 650 million adults globally faced risks related to obesity, and as per the projections of the WHO, this number is expected to double by the year 2030 [81]. The burgeoning epidemic of childhood obesity in contemporary societies has experienced a more than fourfold increase over the last four decades. This trend is not only recognized as a risk factor for adult obesity but also emerges as a primary concern in developing countries [86,87].

Obesity is influenced by a variety of factors, including genetic predisposition, sedentary lifestyle, poor dietary habits, exposure to certain heavy metals, psychological factors, and endocrine disorders [82,88,89]. Ultimately, obesity results from an imbalance between energy intake and expenditure [82]. Obesity gives rise to a spectrum of adverse consequences, including cardiovascular diseases, hypertension, type 2 diabetes, polycystic ovary syndrome, infertility, non-alcoholic fatty liver disease, gastric reflux, musculoskeletal disorders, and an increased risk of certain cancers [82,86,90]. Given the recognized impact of PFAS as one of the contributing factors to obesity, imperative measures are essential for the management of these compounds and the mitigation of human exposure to them [26,91].

### 5.2 Endocrine disrupting

The endocrine system is an intricate network comprising numerous glands distributed throughout the body. Through the secretion of hormones, this system orchestrates growth, development, and the delicate regulation and control of a diverse array of physiological processes [92,93]. However, substances identified as endocrine disruptors have the potential to interfere with the normal functioning of this essential system [68]. According to the Environmental Protection Agency (EPA) definition, endocrine disruptors are agents that interfere with the synthesis, secretion, transport, binding, or removal of natural hormones [94]. The impacts inflicted by endocrine disruptors can manifest in lifelong consequences [68]. Furthermore, these compounds pose a potential threat to the well-being of future generations [68,95].

Pregnant women and children emerge as the most vulnerable demographic groups susceptible to the adverse effects of these compounds [68]. Compounds including phthalates, bisphenols, triclosan, triclocarban, parabens, per- and poly-fluoroalkyl substances, pesticide compounds, and certain drugs, among others, are associated with adverse health consequences. Exposure to these compounds has been linked to conditions such as diabetes, cardiovascular diseases, thyroid dysfunction, birth defects, fertility problems, premature birth, cancer, and various other health issues [66,68,96]. Furthermore, endocrine disorders, both directly and indirectly by fostering adipogenesis, enhancing fat accumulation, and influencing energy balance, contribute to the onset of obesity. This study delves into the intricate connections between endocrine disorders and the diverse spectrum of injuries associated with obesity [97].

### 5.3 PFASs adverse effects

Recent findings propose a potential link between PFASs and the development of dyslipidemia and weight gain [98]. Several studies indicate the capacity of these compounds to disrupt metabolic systems and impact obesity, encompassing phenomena such as the accumulation of fatty liver and alterations in blood lipids. PFAS substances have the capability to alter lipid metabolism, consequently promoting increased fat accumulation within the body [99,100]. During crucial periods of development, these endocrine-disrupting compounds have the potential to induce obesity in early life. This may occur through their effects on the hypothalamus-pituitary-thyroid axis, disrupting thyroid hormones that play a vital role in normal growth and development [101]. Additionally, they can elevate the risk of obesity in children and adolescents by activating PPARγ (peroxisome proliferator-activated receptor gamma) and PPARα (peroxisome proliferator-activated receptor alpha), pivotal factors in lipid and glycogen metabolism [102]. PPARγ and PPARα are both members of the nuclear receptor superfamily of transcription factors. These receptors are essential for controlling several aspects of glucose and lipid metabolism [103,104]. Activation of PPARγ is related to an increase in fat cell production and adipose tissue enlargement. Since this receptor increases insulin sensitivity, it is frequently the target of type 2 diabetes medicines. By encouraging the use of fatty acids as an energy source, PPARα activation can lessen the build-up of fat in tissues and may help regulate metabolic problems linked to obesity. Obesity may result from increased fat accumulation caused by PPARγ activation, particularly when it is dysregulated. Conversely, PPARα activation encourages the metabolism of fat for energy, which may mitigate the metabolic consequences of obesity [105,106].

Exposure to PFASs also manifests in adverse effects on the reproductive system, mediated by the induction of endocrine disorders [36,107]. PFAS substances possess the potential to detrimentally impact the metabolism, production, and secretion of vital steroid hormones, including progesterone, testosterone, estradiol, follicle-stimulating hormone, and luteinizing hormone [36]. Furthermore, by influencing the levels of thyroid-stimulating hormones (FSH), triiodothyronine (T3), and thyroxine (T4), and disrupting thyroid function, PFAS substances can precipitate alterations in reproductive hormones, contributing to menstrual irregularities, early menopause, and infertility in women [36,108]. Exposure to these substances may be linked to conditions such as polycystic ovary syndrome, elevating the risk of diseases including ovarian cancer and endometriosis [36,109].

Epidemiological studies have consistently highlighted a robust association between PFOAs and PFOS, specifically, an increased risk of kidney and testicular cancers [23]. Moreover, PFAS substances can impact the liver, another crucial endocrine organ. As the primary line of defense in the body, the liver is particularly susceptible to the effects of chemicals compared to other organs [64]. Owing to their structural resemblance to fatty acids, PFAS compounds can bind to proteins associated with fatty acids in the liver. Additionally, these substances can integrate into bile, accumulating in the liver and potentially causing liver damage, ultimately heightening the risk of non-alcoholic fatty liver disease [64,110]. The heart, functioning as another endocrine organ, is susceptible to the damage induced by PFAS substances [21]. Table 4 unveiled the impact of PFASs on obesity and endocrine disorders through comprehensive studies.

**Table 4:** PFAS exposure impact on organs, exposure rates, and associated outcomes.

Substance	Exposed Group	Target Organ	Exposure Rate	Outcomes	Ref.
PFOA	Pregnant mothers and children up to age 20	Intrauterine exposure	–	Positive association in girls, no Discernible effect in boys	[164]
PFOA & PFOS	Pregnant mothers and children aged 5-9	Intrauterine exposure	–	A positive association between PFAS exposure and abdominal obesity	[118]
PFOA	Pregnant mice	Intrauterine exposure	0.01 mg/BW	A positive association between PFAS exposure and weight gain	[165]
PFOA & PFNA	Pregnant mothers and male children	Intrauterine exposure	–	Positive association with obesity	[166]
PFOS & PFHxS	Pregnant mothers and female children	Intrauterine exposure	–	Positive association with obesity	[166]
PFOS	Black-spotted frog	–	10 μg/L	Alterations in lipid metabolism, endocrine system, and immune system	[137]
PFOS	Zebrafish	Testicle	<0.2 μM	Elevated expression of thyroid growth-related genes and reduced expression of genes regulating sex hormones	[138]
PFOA, PFOS & PFOSA	Zebrafish	Heart	1 & 10 μg/L	Reduction of heart rate, stroke volume, and cardiac output	[139]
PFOS, PFBA & PFBS	Male rat	Testicle	–	Changes in testosterone levels	[141]
PFOA	Mouse	Pancreas	0.5, 2.5, and 5 mg/BW	Oxidative stress in the pancreas	[146]
PFOS	Zebrafish	Pancreas	16, 32, and 64 μM	Reduction of the islet surface and the overall size of the pancreas	[147]
PFOS & PFDoA	Human	Thyroid	–	Positive and negative relationship with thyroid hormones	[125]
PFOS	Human	Liver	>55 μM	Liver cancer	[128]
PFOA, PFOS & PFHxS	Women aged 20-40	Ovary	–	Primary ovarian failure	[129]
PFOA	Adolescents aged 12-19	Kidney	–	Decreased kidney function and increased uric acid level	[130]
PFOA	Mouse	Kidney	1, 5, 10, and 20 mg/kg	Epigenetic changes in the kidney	[133]

**PFOA:** Perfluorooctanoic acid.

**PFOS:** Perfluorooctane sulfonate.

**PFNA:** Perfluorononanoic acid.

**PFHxS:** Perfluorohexanesulphonic acid.

**PFHxA:** Perfluorohexanoic acid.

**PFHpA:** Perfluoroheptanoic acid.

**PFDoA:** Perfluorododecanoic acid.

**PFBA:** Perfluorobutanoic acid.

**PFBS:** Perfluorobutanesulfonic acid.

**PFOSA:** Perfluorooctanesulfonamid

Research findings underscore the detrimental impact of PFAS compounds on both human health and wildlife, inducing endocrine dysfunction and contributing to obesity through varied mechanisms. While numerous studies have explored the association between PFAS compounds and obesity, a subset of these investigations remains cautious, citing insufficient evidence and emphasizing the multifaceted nature of obesity influenced by several factors [26]. PFAS compounds, known for their pervasiveness in various consumer products, have been associated with multifaceted effects on human health. These compounds are implicated in disrupting lipid metabolism, perturbing thyroid hormones and steroid hormone homeostasis, and even prompting mesenchymal stem cells to differentiate into adipocytes. This unique ability to influence adipogenesis has led to their classification as “obesogens” [111,112].

Notably, PFAS compounds play a concerning role in pediatric health, particularly in contributing to childhood obesity. Studies indicate that these compounds elevate cortisol levels in umbilical cord blood, thereby affecting the delicate balance of the hypothalamus-pituitary-adrenal axis. This disruption in the hormonal milieu can set the stage for obesity development in children [113]. Significantly, a considerable body of biological research has focused on prenatal exposure to PFAS compounds, revealing a correlation with fetal growth disorders and a subsequent increased risk of childhood obesity. These findings underscore the critical importance of understanding the early-life impacts of PFAS exposure, shedding light on potential preventive strategies to mitigate the adverse health outcomes associated with these pervasive environmental contaminants [114].

PFOA, a prominent member of the PFAS family, exhibits the potential to activate peroxisome proliferator-activated receptors (PPARα and PPARγ). These receptors play pivotal roles as key lipid regulators, influencing lipid homeostasis and contributing significantly to adipocyte differentiation, lipid metabolism, and fatty acid oxidation [115]. Additionally, there is an indication that PFOA might impact thyroid hormone homeostasis, a crucial factor in regulating overall energy metabolism. However, it's essential to note that further comprehensive studies are required to validate and deepen our understanding of these associations [116]. Remarkably, studies have unveiled a positive correlation between intrauterine exposure to PFOA and an increased prevalence of overweight in female children at the age of 20. Intriguingly, this relationship wasn't observed in male children. The potential influence of PFOA on ovarian development, leading to the disruption of estrogen synthesis, has been proposed as a mechanistic link to obesity, highlighting the intricate interplay between these compounds and hormonal regulation [117]. Furthermore, investigations have revealed a positive link between prenatal exposure to both PFOA and PFOS and an elevated risk of obesity, particularly around the waist, with a more pronounced impact observed in girls [118,119]. This gender-specific sensitivity implies that women may be more vulnerable to the effects of these compounds compared to men. These findings underscore the complexity of PFAS-related health impacts, emphasizing the need for continued research to unravel the intricacies of these associations and inform targeted interventions [120].

On the other hand, the potential association between PFAS and obesity is believed to stem from disturbances in the signaling pathways of adipokines [121]. Adipokines, a group of proteins secreted by adipose tissue, function as endocrine organs, and irregularities in their secretion are strongly linked to metabolic disorders, including obesity, type 2 diabetes, and cardiovascular diseases. Leptin, adiponectin, resistin, and visfatin are key adipokines, with leptin playing a crucial role in energy homeostasis, regulation of glucose homeostasis, and body weight control [121,122]. Despite the limited number of studies and a lack of comprehensive understanding regarding the mechanisms through which PFAS affects adipokines, alterations in the secretion of adipokines, particularly leptin, have been observed in both human and animal studies. Such changes in adipokine secretion could be considered as one of the potential mechanisms through which PFAS influences obesity. For instance, a study by Hines et al. demonstrated that exposure of mice to a concentration of 0.01 mg/kg of PFOA during pregnancy resulted in increased weight, leptin, and insulin levels in the offspring's mid-life [123]. Additionally, Shelly et al. explored the relationship between PFAS and adipokine hormone levels from childhood to adolescence, highlighting the disruption of human adipokine hormone regulation following PFAS exposure as a potential health consequence. Importantly, the results varied based on gender and age [124].

The impact of intrauterine PFAS exposure on fetal thyroid hormone (TH) levels has been a subject of observation. PFASs characterized by short chains or containing carboxyl groups demonstrate a heightened ability to traverse the placental barrier, posing a potential threat to fetal health. In this study, a noteworthy positive correlation was identified between PFOS and TH3, along with a negative correlation between PFDoA and TH3. Additionally, a significant positive correlation between PFDoA and TH4, as well as a negative correlation of PFAS with thyroid-stimulating hormone (TSH), were observed. The precise mechanism underlying the effects of PFAS on THs remains not entirely clear, but several potential mechanisms can be considered. Firstly, PFAS might competitively bind to thyroid hormone-binding proteins, such as thyroxine-binding globulin (TGB), transthyretin (TTR), and albumins. Secondly, PFAS could potentially elevate the concentration of deiodinase type 1, leading to an increased conversion of T4 to T3. Thirdly, a reduction in TSH levels through the hypothalamus-pituitary-thyroid axis could be another plausible mechanism [125].

Various studies have highlighted the impact of PFAS on thyroid homeostasis, noting its influence on estrogen and other sex hormones. PFAS has been shown to increase estrogen-dependent gene expression and alter concentrations of both estrogen and androgen [125,126]. Moving beyond the endocrine system, the liver—an essential endocrine organ central to homeostasis and metabolic regulation—is also susceptible to the effects of PFAS compounds [127]. High levels of PFOS exposure have been associated with an increased risk of liver cancer, potentially mediated through alterations in glucose, amino, and bile metabolism. PFASs pose a risk factor for Hepatocellular Carcinoma (HCC) by disrupting insulin and glucose regulation, characteristics often seen in type 2 diabetes [128]. Exploring the relationship between per- and polyfluoroalkyl compounds and early ovarian failure, it was found that high exposure to PFOA, PFOS, and PFHXS compounds was linked to an increased risk of Primary Ovarian Insufficiency (POI) in women. PFOA and PFOS, prevalent in the body and subjects of numerous epidemiological studies, have been implicated in ovarian dysfunction and early menopause by suppressing ovarian hormones and disrupting follicular growth [129].

Perfluoroalkyl compounds also exhibit effects on kidney function, contributing to a reduction in kidney function and an increase in uric acid levels in adolescents [130]. Accumulation of PFOAs, particularly in kidneys—the primary organ of PFOA accumulation—is attributed to slow renal excretion. While these compounds induce nephrotoxicity and adverse effects on kidney function, the exact mechanism remains unknown [131]. Animal studies support the impact of PFOS on the kidneys, revealing kidney hypertrophy in exposed mice [132]. Additionally, PFOAs have been implicated in causing epigenetic changes, followed by the stimulation of genes involved in the activation of fibrotic fibroblasts in mice [133].

Certain animal studies suggest the presence of estrogenic activities in some PFAS compounds, indicating their interaction with estrogen receptors (ERs). These receptors hold significant roles in crucial physiological processes such as energy balance, glucose homeostasis, and lipid metabolism. The observed estrogenic effects of PFAS compounds in these studies underscore the potential implications for endocrine disruption and metabolic regulation, adding a layer of complexity to the understanding of the biological impacts of these environmental contaminants [[134], [135], [136]]. An association was identified between PFOS exposure and disruptions in lipid metabolism, immune function, and endocrine glands in black-spotted frogs, culminating in liver damage. Upon entering the organism, these compounds demonstrated an ability to bind with Peroxisome-Proliferator Activated Receptors (PPAR), which are transcription factors responsible for regulating essential processes like lipid and glucose metabolism. The impact of PFOS on PPAR expression was observed in frogs, implicating the PPAR signaling pathway, crucial for lipid metabolism, as a target of PFOS-induced effects. In the conducted study, glycolipid binding was notably and significantly affected by PFOS compounds. Moreover, these compounds exhibited a substantial influence on steroid biosynthesis, steroid hormone biosynthesis, and thyroid hormone synthesis. This intricate disruption of various metabolic and endocrine pathways underscores the comprehensive impact of PFOS on the physiological processes of black-spotted frogs, highlighting potential ecological consequences and the intricate interplay of PFAS compounds with key biological pathways [137].

In a study focused on the toxicity of PFAS compounds on zebrafish, exposure to PFOS at concentrations lower than 0.2 μM was found to increase the expression of genes associated with thyroid growth while simultaneously decreasing the expression of genes regulating sex hormones [138]. Furthermore, exposure to PFOS, PFOA, and PFOSA resulted in a high incidence of cardiotoxicity during the early stages of zebrafish development, as evidenced by RNA-seq, gene expression, and protein level analyses. This cardiotoxicity manifested through an increase in pericardial area, sinus venosus–bulbus arteriosus (SV-BA) distance, and induction of atrial natriuretic peptide (ANP) content, ultimately leading to a decrease in heart rate, stroke volume, and cardiac output. Notably, the impact on reducing cardiac output was more pronounced with PFAS and PFOSA compounds. This study highlighted the potential link between cardiovascular system damage and behavioral changes in fish [139]. Additionally, PFNA and PFBS were identified as causing hepatotoxicity and reproductive, thyroid, and fetal growth dysfunction, along with cardiac abnormalities in zebrafish. PFNA, in particular, was associated with more severe cardiac effects compared to PFBS [116]. In the case of fish (*Perca fluviatilis*) subjected to lifetime exposure to PFAS compounds, adverse effects on the thyroid were observed, impacting T3 levels and the expression of thyroid hormone-related genes. Furthermore, the immune system of the fish was affected by PFAS exposure [140].

A study conducted on male rats unveiled that PFASs possess the capability to influence testicular steroidogenesis. Specifically, exposure to PFOS, PFBA, and PFBS before puberty was found to elevate testicular testosterone concentration [141]. In contrast, other studies have indicated a decrease in the expression of steroidogenic proteins and enzymes subsequent to exposure to PFOA and PFOS [54,142,143]. Furthermore, findings from research on mice suggest that PFOS can activate the biosynthesis of steroid hormones by suppressing histone methylation. Additionally, PFOS has been identified as having the capacity to interfere with the synthesis and secretion of testicular steroid hormones [144].

The pancreas, a central regulator of metabolic function, is identified as another endocrine gland susceptible to damage from PFAS exposure [145]. For instance, PFOAs have been linked to alterations in pancreatic enzyme secretion, focal ductal hyperplasia, and inflammation over a short-term period of 7 days, with long-term exposure leading to the development of pancreatic acinar tumors in mice [146]. Another study indicates that PFOS compounds induce reductions in islet surface and overall pancreatic size, along with a decrease in the expression of digestive genes and glucose-regulating hormones in zebrafish embryos [147]. Furthermore, PFOS has been associated with an increase in fatty acids and a decrease in the expression of PPAR genes. This particular study suggests PFOS as a pollutant with implications for obesity and diabetes [145]. Another investigation highlights the significant impact of PFOA and PFOS, even at very low doses, on the early characteristics of human pancreatic cells [148]. These findings collectively underscore the vulnerability of the pancreas to PFAS-induced damage, potentially influencing metabolic regulation and contributing to the development of various health conditions.

## 6 Cellular and molecular interactions of PFAS in the human body

PFAS compounds, due to their hydrophobic nature, exhibit a high affinity with proteins, particularly albumin, as opposed to fats. This characteristic leads to their extensive binding to proteins, with 90–99 % of these compounds being bound to albumin [108,149,150]. Through blood circulation, PFAS compounds accumulate in various organs, including the kidneys, liver, spleen, brain, and testicles [151]. Furthermore, PFASs with longer carbon chains, such as PFOA and PFOS, possess higher stability and greater potential for accumulation compared to compounds with shorter chains, such as PFBA and PFHxS. The interactions of PFAS compounds with cellular and molecular components within the human body lead to diverse effects on metabolic, endocrine, and reproductive functions [23,36]. These compounds can interfere with hormone metabolism and disrupt the synthesis, secretion, and regulation of various hormones, including thyroid hormones, sex steroids, and adrenal hormones. PFAS exposure has been associated with alterations in thyroid hormone levels, thyroid autoimmunity, and adverse effects on bone metabolism, reproductive health, and adrenal gland function [152,153]. Moreover, PFAS compounds have been implicated in dyslipidemia, hepatotoxicity, renal fibrosis, and disturbances in glucose metabolism, contributing to conditions such as diabetes [154,155].

**Fig. 2:**
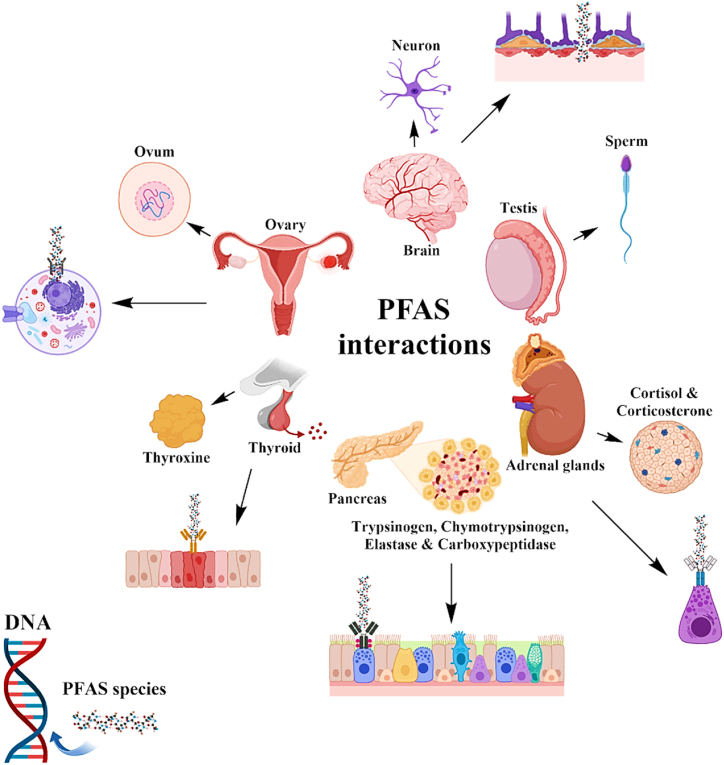
Cellular and molecular interactions of PFASs.

At the cellular level, PFAS compounds can induce oxidative stress, DNA damage, and disruption of hormone receptor signaling pathways. These interactions can lead to dysregulation of cellular processes, including apoptosis, proliferation, and differentiation, ultimately impacting organ function and homeostasis [34,156,157]. Furthermore, PFAS compounds have been shown to modulate gene expression, enzymatic activity, and signaling cascades involved in hormone synthesis, metabolism, and action [158,159]. Overall, the cellular and molecular interactions of PFAS in the human body underscore the complex mechanisms underlying their physiological effects and highlight the need for further research to elucidate their impact on human health and develop effective strategies for mitigation and management (Fig. 2).

## 7 Conclusion and future perspective

Recent studies have revealed a direct correlation between polyfluoroalkyl compounds present in drinking water and the prevalence of obesity and endocrine disorders. Given the pivotal role of the endocrine system in human health and the dire consequences that ensue from its malfunction, the imperative to replace and eradicate these substances cannot be overstated. However, despite efforts to remove these compounds, their transmission through the food chain, coupled with their propensity for accumulation and biological magnification, portends ongoing risks to human health and the environment. Consequently, future research must prioritize the development of strategies for replacing and eliminating these compounds to mitigate their adverse effects. This necessitates a comprehensive approach that acknowledges existing challenges and embraces multidisciplinary collaboration:

1. Gradually phasing out these compounds is essential, although a complete ban may be unfeasible.
2. Replacement efforts necessitate thorough research to prevent substitution with potentially more toxic or poorly understood compounds.
3. Evidence suggests that PFAS compounds with shorter chains exhibit lower accumulation potential, yet comprehensive studies on these compounds remain insufficient. Further scrutiny of understudied or unidentified compounds is imperative to substantiate this assertion.
4. With the increasing substitution of long-chain compounds with short-chain alternatives, the likelihood of encountering these compounds in drinking water is expected to rise, underscoring the need for effective mitigation measures.
5. Some topics addressed in this study warrant additional investigation due to limited or conflicting findings.
6. The existing data on the role of these compounds in activating PPARα and PPARγ is sparse and warrants further exploration.
7. Understanding the effects of PFAS compound mixtures is crucial for comprehensively assessing their impact on human health. Therefore, research efforts should prioritize this aspect as well.
8. Considering the aforementioned points, alongside variations in compound concentrations globally and disparate exposure levels among different populations, the development of national and regional guidelines is imperative.

## CRediT authorship contribution statement

**Hoda Pezeshki:** Writing – review & editing, Writing – original draft, Validation, Methodology, Investigation, Formal analysis, Data curation, Conceptualization. **Saeed Rajabi:** Writing – review & editing, Writing – original draft, Visualization, Validation, Supervision, Software, Resources, Project administration, Methodology, Investigation, Funding acquisition, Formal analysis, Data curation, Conceptualization. **Majid Hashemi:** Writing – original draft, Validation, Methodology, Investigation, Funding acquisition, Data curation, Conceptualization. **Saeideh Moradalizadeh:** Writing – original draft, Investigation, Formal analysis, Conceptualization. **Habibeh Nasab:** Writing – original draft, Investigation, Formal analysis, Conceptualization.

## Data availability

No new data was generated for the research described in the article.

## Declaration of competing interest

The authors declare that they have no known competing financial interests or personal relationships that could have appeared to influence the work reported in this paper.
